# Clinical significance and diagnostic usefulness of serologic markers for improvement of outcome of tonsillectomy in adults with chronic tonsillitis

**DOI:** 10.1186/1477-5751-12-11

**Published:** 2013-07-01

**Authors:** Silvia Bohne, Robert Siggel, Svea Sachse, Michael Kiehntopf, Michael Bauer, Eberhard Straube, Orlando Guntinas-Lichius

**Affiliations:** 1 Department of Otorhinolaryngology, Jena University Hospital, Jena, Germany; 2 Institute of Medical Microbiology, Jena University Hospital, Jena, Germany; 3 Institute of Clinical Chemistry and Laboratory Diagnostics, Jena University Hospital, Jena, Germany; 4 Center for Sepsis Control and Care (CSCC), Jena University Hospital, Jena, Germany

**Keywords:** Serology, Outcome, Prognostic Marker, Peritonsillar Abscess, Chronic Tonsillitis

## Abstract

**Background:**

The aim of the present study was to explore serological biomarkers which predict the outcome of tonsillectomy for chronic tonsillitis.

**Methods:**

A case study in a University ENT department of 24 adult patients with chronic tonsillitis (CHT) in comparison to 24 patients with acute peritonsillar abscess (PTA) was performed. Blood samples for clinical routine hematological and serological parameters were assessed prior to surgery (T-1) and five days (T5) after tonsillectomy. Outcome 6 months later (T180) was documented using the Glasgow Benefit Inventory (GBI) and the Specific Benefits from Tonsillectomy Inventory (SBTI). Correlation analyses between CHT and PTA group as well as between the different time points within each group concerning the serological parameters and the outcome parameters were performed.

**Results:**

At T-1, patients in the CHT group presented with significantly higher lymphocytes counts (relative and absolute), basophils (relative and absolute) and eosinophils but less white-cells, monocytes, neutrophils (absolute and relative), alpha-1, alpha-2, beta globulins, immunoglobulin and lower C-reactive protein and procalcitonin values than patients in the PTA group (all p < 0.05, respectively). Within each group, different significant changes of the serum parameters (often in opposite direction) were observed between T-1 and T5. SBTI scores at T-1 were significantly lower in the CHT group. In contrast, most GBI scores at T180 were significantly higher in the CHT group. Between T-1 and T180 the SBTI scores improved in three quarters of the CHT patients but only in three fifths of the PTA patients. Higher eosinophil counts and immunoglobulin E levels at T-1 predicted higher GBI scores at T180 in the CHT group.

**Conclusions:**

This pilot study showed a specific serological pattern for patients with chronic tonsillitis with a specific pattern of changes after tonsillectomy. But there is no established role for biomarkers currently used in clinical practice to predict the outcome of tonsillectomy for chronic tonsillitis.

## Background

Chronic tonsillitis (CHT) is one of the most frequent otolaryngologic diseases. The standard therapeutic approach is tonsillectomy [1]. The widely accepted criteria for surgery are at least 3–7 episodes of tonsillitis per year despite medical therapy, but these criteria have been arrived arbitrarily and there is no international consensus [2]. It has been criticized that these clinical criteria are too superficial and subjective. For instance, worsening or improvement over time is not assessed [3]. Furthermore, the severity of the systemic reaction that might be beyond this chronic focal inflammation is not specified yet and taken into account for decision making for surgery. Nevertheless, even with limited criteria for patient selection for tonsillectomy the rates of satisfaction with the outcome following surgery are high [4-6]. Although a frequent illness, the literature on surgery for recurrent tonsillitis is limited, especially on surgery in adults [1]. In adults only one small randomized trial with 70 patients and several methodological limitations was performed showing that adults with proven recurrent streptococcal pharyngitis benefit from tonsillectomy in comparison to watchful waiting at 90 days [7,8]. Any advantages of surgery must be balanced against disadvantages. Although rare, tonsillectomy can be associated with life threatening complications such as (major bleeding or sepsis resulting in a mortality rate between one of 15,000-35,000 procedures [8,9].

Thus, the question arises whether other and objective indicators in patients with CHT might meet the demand to better select patients for tonsillectomy. Recently, an index of tonsillitis was proposed including the factors tonsillitis episodes, morbidity period, presence of sclerotic signs like tonsillar sclerosis, scar tissue formation and obstruction of tonsillar crypts, and assessment of streptococcus pyogenes in cultures or by PCR [10]. Clinical studies validating the effectiveness of this index to predict the outcome is, however, lacking.

The palatine tonsil is a major part of Waldeyer’s ring and part of the mucosal immune system. Even in adults with involution of the organ the tonsil hosts important amounts of immunoglobulin-producing cells [11]. The effects of chronic tonsillitis or of tonsillectomy on this regional immune system or the systemic immunoregulatory effects and their influence in the well-being of the patients are not well described.

In a first approach to 1) characterize the systemic response of adult patients with CHT, 2) to describe the effect of tonsillectomy on this systemic response, and 3) to analyze if the systemic response can predict the outcome of tonsillectomy for CHT, we analyzed in a clinical study a large set of serologic markers prior to and after tonsillectomy in relation to the clinical outcome 6 months later using the Glasgow Benefit Inventory (GBI) and the Specific Benefits from Tonsillectomy Inventory (SBTI). As control group we have chosen adult patients with acute peritonsillar abscess (PTA) planned for bilateral tonsillectomy to rule out confounding effects by the surgery itself.

## Methods

### Design

A prospective clinical cohort study was performed at the Department of Otorhinolaryngology, University Jena, Germany. Institutional review board approval by the ethics committe of the Jena University was obtained prior to study initiation. Each patient entering the study signed consent.

### Subjects

Adult patients (18 to 80 years of age) with chronic tonsillitis (CHT) admitted for tonsillectomy were included. Patients with unilateral peritonsillar abscess (PTA) and indication for tonsillectomy served as controls. All PTA patients received a bilateral tonsillectomy a chaud, i.e., surgical removal of the tonsils during acute inflammation. This type of control group was chosen (and preferred to a control group of healthy patients) to control the surgical procedure itself as a confounding factor on serologic changes and the functional outcome after surgery. Chronic tonsillitis was defined clinically as chronic infection of the palatine tonsils on the basis of recurrent tonsillitis normally without severe symptoms. Because of this, the patient had to present a history of recurrent intake of antibiotics. The tonsils could have been atrophic as well as hypertrophic. Recurrence implied more than two distinct episodes in a 12-month period, and chronicity a period longer than three months. Peritonsillar abscess was defined as a painful pus collection of infected material in the area of and around the tonsils as complication of an acute severe tonsillitis. The pus collection was confirmed by aspiration. Patients with other chronic inflammatory diseases, coagulation disorders, or malignant tumors were excluded. The study enrolment period was from July to December 2009. Fifty-two patients (26 patients for the study group and for the control group, respectively) fulfilled the inclusion criteria. Four patients (2 patients for the study group and for the control group, respectively) had to be excluded because of decline of participation. The amount of episodes of tonsillitis with the last 12 months prior to admission was documented for all patients.

All patients underwent a cold steel dissection of both palatine tonsils. Hemostasis was achieved by bipolar coagulation. All patients received antibiotics perioperatively, because perioperative antibiotic treatment reduces postoperative pain [12]. At the day of surgery (T0), a biopsy of one tonsil (in case of peritonsillar abscess of the affected side) was taken for microbiological investigations. The bacteriological culture was performed for standard bacteriological analysis. The tonsillectomy specimens were forwarded for routine histopathological investigation.

### Outcome assessment

Each patient completed the Specific Benefits from Tonsillectomy Inventory (SBTI), questions 1 to 6 the day before surgery (T-1) and all eight questions of the SBTI 180 days after surgery (T180). Furthermore, all patients completed the Glasgow Benefit Inventory (GBI) at T180. At T-1 the patients answered the questionnaires in the hospital At T180 the patients received the questionnaires by regular mail and business reply envelope. The GBI measures patient benefit and was developed especially for otolaryngological interventions [13]. In scoring the GBI, the responses to all 18 questions were averaged so that all questions have equal weight. In addition to the combined total GBI scores for quality of life changes after tonsillectomy, the GBI subscale scores for general benefit, social support and physical benefit were calculated. The SBTI is a modified version of the GBI to measure specific symptom responses to tonsillitis and tonsillectomy [5]. Typical symptoms accompanying chronic tonsillitis were summarized under the scale ‘symptom change’ (questions 1–3). The ‘reduced use of resources’ scale comprises items such as antibiotic use, doctor visits, sick leaves (questions 4–6). These two scales provide five possible choices: a lot more frequently, more frequently, the same as before, less frequently, a lot less frequently. The impact from tonsillectomy on general health and quality of life (QOL) is assessed by the scale ‘general benefit’ (Questions 7 and 8). Choices of answers per item were: extremely positive, positive, no change, negative, extremely negative. The average scores from both inventories (GBI and SBTI) were then transposed onto a continual benefit scale ranging from -100 to +100. A score of -100 indicates maximal negative benefit, a score of 0 indicates no benefit at all, and a score of +100 indicates maximal positive benefit to the patient’s quality of life.

### Serology

Blood samples for routine hematological and serological tests as well as for aerob and anaerob microbiological culture analysis were taken at T-1 and five days after surgery (T5).

### Statistics

We used IBM SPSS Statistics 19.0.0 for statistical analyses. Data is presented as means ± standard deviation if not otherwise indicated. Chi-square test and Mann–Whitney *U* test for independent samples were performed to analyze differences between the CHT group and the control group of PTA patients at each time point (Tables 1, 2 and 3). Within each group, in the CHT and separately in the PTA group, the correlation of serology parameters at T-1 with the functional outcome at T180 was examined with via Pearson product–moment correlations. In reference to the results of previous studies [6], the four most important functional outcome subscales (GBI social subscale; GBI physical subscale; SBTI resources subscale; SBTI benefit subscale) were selected for multiple comparisons (Additional file 1: Table S1 and Additional file 2: Table S2). The Bonferroni correction was used to adjust the P value while performing multiple comparisons: As four outcome subscales were examined, the level of significance was set at p < 0.0125 (=0.05/4). The Wilcoxon test for dependent samples was used to analyze differences between the time points T-1 and T5 or T180 within each study group (Additional file 3: Table S3). All tests (with exception of the correlation analysis, see above) were performed 2-tailed and conducted at a p < 0.05 significance level.

**Table 1:** Comparison of serologic parameters in patients with chronic tonsillitis to patients with peritonsillar abscess at the day before tonsillectomy (T-1)

**Parameter**	**Chronic tonsillitis**		**Peritonsillar abscess**		p**
	**Mean**	SD*	**Mean**	SD*	
Antistreptolysin O titer (IU/mL)	151.38	164.42	207.24	299.22	0.941
Basophils (Differential count; %)	0.42	0.23	0.17	0.093	**0.000**
Basophils(Gpt/L)	0.04	0.027	0.03	0.025	**0.023**
Alpha-1 globulin (SPEP; %)	4.19	0.77	5.83	1.10	**0.000**
Alpha-2 globulin (SPEP; %)	9.46	1.46	12.06	1.55	**0.000**
Albumin (SPEP; %)	58.04	3.54	53.11	3.19	**0.000**
Beta Globulin (SPEP; %)	11.58	1.89	12.75	1.36	**0.005**
Gamma Globulin (SPEP; %)	16.73	1.84	16.03	2.82	0.435
C-reactive protein (mg/L)	3.68	6.30	99.06	61.63	**0.000**
Eosinophils (Differential count; %).	2.14	1.83	0.51	0.62	**0.000**
Eosinophils (Gpt/L)	0.14	0.10	0.06	0.074	**0.001**
Red-cell count	4.68	0.36	4.80	0.43	0.389
Hemoglobin (mmol/l)	8.45	0.76	8.83	0.86	0.153
Hematocrit	0.41	0.03	0.42	0.04	0.259
Immunoglobulin A (g/L)	2.23	1.74	2.80	0.96	**0.007**
Immunoglobulin E (kU/L)	117.71	186.42	92.88	105.28	0.468
Immunoglobulin G (g/L)	12.69	2.04	12.18	2.63	0.302
Immunoglobulin M (g/L)	1.03	0.53	1.15	0.52	0.444
White-cell count	7.21	1.66	12.49	3.58	**0.000**
Lymphocytes (Differential count; %)	31.99	11.70	14.90	7.23	**0.000**
Lymphocytes (Gpt/L)	2.22	0.72	1.73	0.61	**0.009**
Mean corpuscular hemoglobin (fmol)	1.81	0.11	1.84	0.08	0.932
Mean corpuscular hemoglobin concentration (mmol/l)	20.72	0.53	20.98	0.42	0.082
Mean corpuscular volume (fL)	87.34	4.54	87.73	3.17	0.758
Monocytes (Differential count; %)	7.00	1.67	8.40	3.20	**0.038**
Monocytes (Gpt/L)	0.49	0.14	1.04	0.44	**0.000**
Neutrophils (Gpt/L)	4.30	1.68	9.70	3.44	**0.000**
Neutrophils (Differential count; %)	58.46	12.67	76.02	8.66	**0.000**
Procalcitonin (ng/mL)	0.07	0.03	0.10	0.04	**0.033**
Red Blood Cell Distribution Width (%)	13.28	1.05	13.27	0.76	0.684
Platelet count (Gpt/L)	290.79	48.49	280.74	79.03	0.587
Proteins, total (g/L)	77.83	6.10	78.01	5.35	0.401

*SD standard deviation, **p p value due to Mann–Whitney *U* test; significant p values (<0.05) in bold; *IU* International Unit, *Gpt/L* 10^9^ cells per liter, *SPEP* Serum protein electrophoresis.

**Table 2:** Comparison of serologic parameters in patients with chronic tonsillitis to patients with peritonsillar abscess 5 days after tonsillectomy (T5)

**Parameter**	**Chronic tonsillitis**		**Peritonsillar abscess**		p**
	**Mean**	SD*	**Mean**	SD*	
Antistreptolysin O titer (IU/mL)	138.46	162.15	269.48	360.14	0.398
Basophils (Differential count; %)	0.37	0.16	0.33	0.14	0.572
Basophils(Gpt/L)	0.03	0.02	0.03	0.011	0.736
Alpha-1 globulin (SPEP; %)	5.47	0.70	5.52	0.73	0.897
Alpha-2 globulin (SPEP; %)	11.25	1.23	12.05	1.10	**0.026**
Albumin (SPEP; %)	55.64	2.96	53.21	2.73	**0.005**
Beta Globulin (SPEP; %)	11.89	1.97	12.13	1.21	0.192
Gamma Globulin (SPEP; %)	15.75	1.84	17.10	2.94	0.105
C-reactive protein (mg/L)	16.81	13.66	15.37	12.23	0.639
Eosinophils (Differential count; %).	2.46	1.45	2.17	1.52	0.417
Eosinophils (Gpt/L)	0.17	0.11	0.17	0.10	0.751
Red-cell count	4.39	0.47	4.60	0.45	0.121
Hemoglobin (mmol/L)	7.90	0.90	8.41	0.89	0.088
Hematocrit	0.38	0.04	0.40	0.04	0.149
Immunoglobulin A (g/L)	2.20	1.71	2.83	0.92	**0.004**
Immunoglobulin E (kU/L)	140.06	217.78	87.36	102.88	0.803
Immunoglobulin G (g/L)	11.67	1.83	12.45	3.34	0.823
Immunoglobulin M (g/L)	0.89	0.45	1.24	0.48	**0.005**
White-cell count	7.26	1.70	7.85	1.98	0.318
Lymphocytes (Differential count; %)	25.63	7.29	26.75	9.45	0.489
Lymphocytes (Gpt/l)	1.84	0.59	2.06	0.75	0.245
Mean corpuscular hemoglobin (fmol)	1.80	0.11	1.83	0.08	0.733
Mean corpuscular hemoglobin concentration (mmol/L)	20.63	0.65	20.97	0.45	**0.014**
Mean corpuscular volume (fL)	87.30	4.27	87.15	3.48	0.255
Monocytes (Differential count; %)	7.51	2.54	7.51	1.99	0.751
Monocytes (Gpt/L)	0.54	0.02	0.59	0.21	0.360
Neutrophils (Gpt/L)	4.68	1.34	5.01	1.66	0.542
Neutrophils (Differential count; %)	64.03	7.83	63.24	10.04	0.589
Procalcitonin (ng/mL)	0.06	0.03	0.07	0.02	0.065
Red Blood Cell Distribution Width (%)	13.16	1.14	12.87	0.62	0.403
Platelet count (Gpt/L)	285.95	59.86	324.91	75.84	0.209
Proteins, total (g/l)	73.66	5.63	74.53	6.60	0.639

*SD standard deviation, **p p value due to Mann–Whitney *U* test; significant p values (<0.05) in bold; *IU* International Unit, *Gpt/L* 10^9^ cells per liter, *SPEP* Serum protein electrophoresis.

**Table 3:** Results of the Glasgow Benefit Inventory (GBI) and of the Specific Benefits from Tonsillectomy Inventory (SBTI)

**Score**	**Chronic tonsillitis**		**Peritonsillar abscess**		p**
	**Mean**	SD*	**Mean**	SD*	
GBI total score (T180)	24.2	20.9	7.8	15.5	**0.013**
GBI subscores (T180)					
General health	25.6	21.4	7.0	17.0	**0.009**
Social support	6.7	20.5	2.1	5.7	0.965
Physical functioning	45.2	42.2	16.7	32.2	**0.013**
SBTI (T-1)					
Total score	-3.0	37.0	52.7	39.0	**<0.0001**
Symptom score	-0.8	32.7	47.0	44.7	**<0.0001**
Resources	-5.3	46.7	68.3	37.4	**<0.0001**
SBTI (T180)					
Total score	68.8	41.3	67.9	34.3	0.571
Symptom score	66.7	40.8	62.2	33.0	0.412
Resources	72.5	43.7	76.2	39.1	0.891
Benefit	61.9	32.2	40.6	31.5	0.059
SBTI (ΔT***)					
Total score	74.12	68.77	22.44	57.47	**0.030**
Symptom score	67.50	63.63	20.24	58.90	**0.049**
Resources	81.58	76.95	23.08	59.14	**0.024**
Absolute number of patients with changes of SBTI score between T-1 and T180 (ΔT***)	N****		N****		p**
Total score					0.132
Improved	15		7		
Unchanged/deteriorated	4		6		
Symptom score					0.150
Improved	16		8		
Unchanged/deteriorated	4		6		
Resources					0.146
Improved	16		8		
Unchanged/deteriorated	3		5		

*SD standard deviation; **p p value due to Mann–Whitney *U* test; significant p values (<0.05) in bold; ***the scores at T108 were subtracted from the values at T-1; ****the sample size N could be smaller than 24 as not all patients answered the SBTI T180 or answered all questions.

## Results

Twenty-four patients with chronic tonsillitis (CHT) and twenty-four patients with peritonsillar abscess (PTA) were included. The sex ratio was significantly different in both groups (p = 0.006): The CHT group was dominated by female patients (18 women, 6 men), and vice versa the PTA group (6 women, 18 men). The patients were significantly younger (p = 0.001) in the CHT group (average: 29 years; range: 19–59 years) than in the PTA group (average: 39 years; range: 20–70 years). The number of allergic patients was not different in the groups (p > 0.05) No allergic patient had allergy symptoms between T-1 and T5. On average significantly more tonsillitis episodes within the last 12 months were reported in the CHT group in comparison to the PTA group (CHT group: average: 3.9; range: 3–15 versus PTA group: average 0.8; range: 0–3; p < 0.0001).

Histopatholology confirmed the diagnosis of chronic tonsillitis or peritonsillar abscess, respectively, in all cases. Blood cultures were negative in all but 2 asymptomatic cases: In one patient of the CHT group *Micrococcus lylae* was observed once in a blood sample at T1 and in one patient of the PTA group *Streptococcus epidermidis* once at T5. Microbiology of the tonsils revealed mixed infections in most samples. No pathogens were found in two patients in the CHT group and in one patient of the PTA group. In the CHT group the dominant pathogens (> 2 cases) were: *Streptococcus viridans* (18 cases), *Staphylococcus aureus* (8 cases), *Neisseria spp*. (7 cases), and *Streptococcus pyogenes* (3 cases). In the PTA group the most important pathogens were: *Streptococcus viridans* (14 cases), coagulase-negative staphylococci (7 cases), *Neisseria spp*. (5 cases), and *Staphylococcus aureus* (4 cases).

Blood test results at T-1 and T5 are presented in Tables 1, 2 and Additional file 3: Table S3, respectively. At T-1, patients in the CHT group presented with significantly higher relative and absolute lymphocytes counts than patients in the PTA group (p < 0.0001 and p = 0.009, respectively). Moreover, CHT patients had higher relative and absolute basophils and eosinophils (p < 0.001 and p = 0.023), and higher albumin values (p < 0.0001). In contrast, patients with PTA had higher white-cell numbers (p < 0.0001), more monocytes (p < 0.0001), more neutrophils (absolute and relative; p < 0.0001 and p < 0.0001), more alpha-1, alpha-2 and beta globulins (p < 0.0001; p < 0.0001; P = 0.005), higher immunoglobulin A values (p = 0.0007) and higher C-reactive protein (p < 0.0001) and procalcitonin values (p = 0.033). At T5, fewer differences between the CHT and the PTA group were observed. Only albumin remained higher in the CHT group than in the PTA group (p = 0.005). In the PTA group immunoglobulin A and immunoglobulin M as well as the mean corpuscular hemoglobin concentration were higher (p = 0.004, p = 0.005 and p = 0.014, respectively).

Within each group, different changes of the serum parameters between T-1 and T5, and these changes often in opposite direction, were observed (Additional file 3: Table S3). There was a significant different shift in the serum protein electrophoresis: Albumin and gamma globulin relatively decreased and the other proteins of the serum electrophoresis increased after surgery in the CHT group whereas albumin and gamma globulin relatively increased and the other proteins increased in the PTA group. C-reactive protein increased in the CHT but decreased in the PTA group. Procalcitonin decreased in both groups, but more prominent in the PTA group. The immunoglobulins A, G and M only decreased in the CHT group. Looking on the differential count, there was a shift from relatively decreasing lymphocytes to relatively increasing neutrophils in the CHT group and vice versa in the PTA group. In parallel, an absolute decrease of lymphocytes and increase of neutrophils accompanied by an absolute decrease of platelets in the CHT group and vice versa in the PTA group was observed. Due to surgery and concomitant blood loss, a significant decrease of all directly related parameters (red cells, hemoglobin, hematocrit and others) was seen in both groups.

At T-1 prior to surgery all Specific Benefits from Tonsillectomy Inventory (SBTI) scores were significantly lower in the CHT than in the PTA group (Table 3). At T180 the return rate of the questionnaires was better in the CHT group than in the PTA group: 21 of 24 patients (86%) answered at T180 whereas only 15 of 24 patients (63%) responded in the PTA group. No bias by the non-responders was seen in the CHT group: age, gender, number of tonsillitis episodes, all SBTI scores at T-1 were not different between responders and non-responders (all p > 0.05). In contrast, the age of non-responders in the PTA group was lower than of the responders (median: 30 years versus 44 years, p = 0.025). After surgery and re-evaulation at T180 these differences disappeared. This was mainly related to the significant increase of all SBTI scores within the CHT group (all p < 0.002). The SBTI scores did not significantly increase after surgery in the PTA group (all p > 0.05). All Glasgow Benefit Inventory (GBI) scores (with exception of the social support score) at T180 were significantly higher in the CHT group than in the PTA group. Three quarters of the patients showed an improvement in the SBTI scores after surgery in the CHT group, whereas only about half of the patients in the PTA showed such an improvement. Neither at T-1 nor at T180 most SBTI scores and GBI scores were influenced by age or gender of the patients (all p > 0.05). Only the SBTI resource score at T180 was significantly higher in female than in male patients in the CHT group (p = 0.022).

A few serology parameters at T-1 were significantly associated with the function outcome at T180 as assessed by the GBI and SBTI questionnaires (Additional file 1: Table S1 and Additional file 2: Table S2). In both the experimental and in the control group the influence of the preoperative serology markers did not give a unique pattern for GBI and SBTI scores half a year after surgery. In the CHT group (Additional file 1: Table S1), relative more eosinophils (r = 0.764; p <0.001) as well as absolute more eosinophils (r = 0.642; p = 0.003) and more immunoglobulin E (r = 0.712; p < 0.001) were correlated to a better GBI social score. Moreover, a preoperative lower red cell counts was related to a significant higher GBI physical score (r = -0.546; p = 0.011) six months after tonsillectomy. A significant correlation between serologic markers prior to surgery and the STBI outcome parameters was not seen.

In the PTA group (Additional file 2: Table S2), there was only one significant correlation between pre-surgical serology and functional outcome six months later: A lower initial platelet count was related to a higher STBI resources subscale (r = -0.676; p = 0.011)

## Discussion

Using retrospective studies it is thought that up to 90% of adult patients with chronic tonsillitis profit from tonsillectomy [4]. Using the GBI and the SBTI the present study shows significant benefits for tonsillectomy. This is in accordance with other studies using these questionnaires [5,14]. But even using these standardized assessments GBI and SBTI in a prospective study might overestimate the efficacy because a bias by the surgery itself cannot be ruled out. This surgical bias might could have been especially high in the PTA group as the acute disease phase is extremely painful and the relief by tonsillectomy accordingly high. Therefore, it was very important to set the endpoint of the present study in sufficient distance to the surgery, i.e. 180 days later. The GBI in total and most questions of the SBTI consider the efficacy in a retrospective perspective. To overcome this problem, we used a control group with another disease but same surgery. Furthermore, we asked the applicable questions of the SBTI prior and after tonsillectomy. By this, we clearly revealed that about three quarters of the patients with chronic tonsillitis profit from tonsillectomy and this cannot be attributed to the surgery itself. Otherwise, up to one quarter of patients seems not to profit significantly. Due to ‘The Information System of the Federal Health Monitoring’ about 70,000 tonsillectomies were performed 2009 in adults in Germany (http://www.gbe-bund.de/). Taken into account tonsillectomies for other reasons, it could be assumed that tonsillectomy for chronic tonsillitis was not effective in terms of functional benefit in about 15,000 cases. For the USA, of course, a higher number could be estimated. Actually, this high number should force us to improve the selection criteria for adult tonsillectomy. We should look beyond clinical criteria. Apparently, clinical criteria are insufficient predictors for a good functional outcome after tonsillectomy.

The present study has some methodological limitations. The sample size was small. Anyhow, the effort was already large with several blood samples at definitive time points and a long follow-up of 180 days. The serologic markers and results of surgery-specific quality of life assessments showed a high variability. Additionally, as expected there was an imbalance in the primary functional outcome: the group of satisfied patients was much larger than the group of unsatisfied patients (relation 4:1). Despite of this, several robust statistical results have been observed. Nevertheless, due to the small sample sizes a multivariate analysis would not be meaningful and hence was omitted. There is no consensus how to determine the outcome of tonsillectomy. The only randomized trial on the effectiveness of tonsillectomy compared to watchful waiting for chronic tonsillitis has been criticized for its short follow-up of six months because it could not been ruled out that the effect of tonsillectomy would not be reduced if the follow-up was longer [7,8]. Furthermore, to use the number of episodes of sore throats as primary outcome criterion was criticized as this criterion is difficult to define and to standardize. In the present study an identical follow up period of six months was used but the surgery-specific quality of life assessments GBI and SBTI were applied to determine the functional outcome. The GBI has been proven to be a valid instrument to study changes of the health status after otolaryngological surgery including tonsillectomy [14]. Because the GBI-items are of a general nature to address postoperative changes, items of SBTI were specifically designed to assess only the symptoms of chronic tonsillitis [5]. Both scores seem to be much more sensitive than to count episodes of sore throat or tonsillitis to evaluate the impact of tonsillectomy. Nevertheless, due to the non-specific (GBI) versus specific design for tonsillectomy (SBTI) and because of the different perspective on the surgical procedures (GBI: retrospective versus SBTI: prospective), both scores are not directly comparable [5]. This became obvious in a recent study and can be confirmed by the present investigation [5].

The serologic investigations revealed several principal results: First, the serologic profiles form patients with CHT differ from patients with PTA before surgery (T-1) and partly opposite changes of these profiles can be observed quickly after surgery (T5). At a first glance, this should be taken for granted as the first group of patients is suffering from a chronic and gradual inflammatory disease whereas the second group suffers from an acute abscess formation due to the aggressiveness of a local bacterial infection. This should explain significantly higher values for parameters related to hemogram and positive (e.g. c-reactive protein) as well as negative acute phase reactant (e.g. albumin) indicating acute inflammation in the PTA group prior to surgery and their drop down after surgery (much higher c-reactive protein, higher procalcitonin, more white cells, shift to relative more neutrophils in the differential count and less of the other white cell types). In addition, there is the significant shift in the serum protein electrophoresis from relative more albumin in the CHT group to alpha-1 and alpha-2 globulin in the PTA group. The explanation for the shift might be that the alpha globulins include several acute phase proteins indicating acute inflammation. More interesting are the significant lower immunoglobulin A (IgA) values in the CHT group. The tonsils are part of the MALT system (Mucosal associated lymphoid tissues). The MALT system as part of the secondary lymphoid tissue is one of the first defense barriers against microorganisms. T and B lymphocytes in the tonsils are mainly secreting IgA [11]. Although decreasing with age this production is still relative high in healthy adults [11]. It is well known that chronic tonsillitis leads to a decrease of IgA by its destruction of the functional tonsil [11]. Moreover, a secretory IgA deficiency is observed in tonsillectomized subjects [15]. But actually this observation is exclusively based on postoperative data not taking into account that the deficiency might have been established, at least partly, already prior to surgery as part of the chronic inflammation of the tonsil. We speculate that a lower level of IgA might be the expression of a more advanced destruction of the tonsil by the chronic inflammation. For the next investigations we plan to measure also the salivary IgA level. This would give a further hint on a modulated immune defense in patients with chronic tonsillitis.

Looking on potential prognostic markers for the predication of better functional outcome, only a few significant correlations between serologic markers and functional outcome was observed in the CT group but only for one parameter in the PTA group. A consistent and easy to explain pattern of markers was not found. This might support the hypothesis that chronic tonsillitis as a matter of fact has systemic effects which can be counteracted by tonsillectomy. Interestingly, higher eosinophil cell levels and a higher immunoglobulin E level (additionally, eosinophil levels and immunoglobulin E level were highly correlated to each other, data not shown) were correlated to better functional outcome. A correlation to the atopy status of the patients was not evident, but the sample sizes might be too small to detect such an association. There is some controversy if there is correlation between atopy and adenotonsillar hypertrophy in children and a localized tonsillar allergic reaction is also discussed [16]. Anyhow, up to the present no data on this question is available for adults with chronic tonsillitis. It is well known that eosinophils are part of the chronic inflammatory process in the tonsils [17]. Furthermore, eosinophils are highly activated and responsive to different inflammatory stimuli in children with chronic tonsillitis [18]. But so far eosinophils were not considered as a potential serologic prognostic marker for outcome after tonsillectomy. In agreement with the present results, it has been shown also for children with chronic tonsillitis that serum IgE concentrations are changed by tonsillectomy [19]. To proof the hypothesis that eosinophils and its related inflammatory mediators might play more important role in chronic tonsillitis it is planned not only to repeat the study with a larger sample size to confirm the findings but also to add a genomic and proteomic expression analysis with focus on the eosinophil granulocyte function.

## Conclusions

This study revealed that patients with chronic tonsillitis show specific patterns of serologic parameters. These parameters change immediately after tonsillectomy. The patterns and changes are significantly different from patients with acute peritonsillar abscess. Nevertheless, due to the present pilot study it appears that the preoperative serologic biomarkers to not clearly predict the outcome of tonsillectomy for chronic tonsillitis. We propose to extend the research program by gene expression profiling of the blood and tonsillar tissue of the patients.

## Abbreviations

CHT: Chronic tonsillitis; GBI: Glasgow Benefit Inventory; PTA: Acute peritonsillar abscess; SBTI: Specific Benefits from Tonsillectomy Inventory.

## Competing interests

The authors declare that they have no competing interests.

## Authors’ contributions

MK, MB, ES, and OGL completed the study design. OGL drafted the manuscript. SG revised the manuscript for intellectual content. SS and ES completed the microbiological analysis. MK completed the serological analysis. SB and RS completed the acquisition of the clinical data. OGL and RS completed statistical data analysis. In addition, all authors have seen and approved the final version of this manuscript for submission.

## Supplementary Material

Additional file 1: Table S1 Correlation between of preoperative serology (T-1) and functional outcome at T180 in the group of patients with chronic tonsillitis (CHT); r and p-values*. Click here for file

Additional file 2: Table S2 Correlation between of preoperative serology (T-1) and functional outcome at T180 in the group of patients with peritonsillar abscess (PTA); r and p-values*. Click here for file

Additional file 3: Table S3 Changes of serology within each group of patient between T-1 and T5. Click here for file
