# Supplementary material for: Clinical significance and diagnostic usefulness of serologic markers for improvement of outcome of tonsillectomy in adults with chronic tonsillitis
Source: J Negat Results Biomed. 2013 Jul 1;12:11. doi: 10.1186/1477-5751-12-11 (PMC3701599; doi:10.1186/1477-5751-12-11)
Supplement: Additional file 1: Table S1 — Correlation between of preoperative serology (T-1) and functional outcome at T180 in the group of patients with chronic tonsillitis (CHT); r and p-values*. [file 1477-5751-12-11-S1.doc]

**Additional file 1: Table S1. Correlation between of preoperative serology (T-1) and functional outcome at T180 in the group of patients with chronic tonsillitis (CHT); r and p-values***

| Serology at T-1 | GBI  T180  Social | | GBI  T180  Physical | | SBTI  T180  Resources | | SBTI  T180  Benefit | |
| --- | --- | --- | --- | --- | --- | --- | --- | --- |
| Parameter | r | p | r | p | r | p | r | p |
| Antistreptolysin O titer (IU/mL) | -0.14 | 0.567 | 0.252 | 0.285 | 0.204 | 0.389 | 0.406 | 0.076 |
| Basophils (Differential count; %) | 0.408 | 0.083 | 0.177 | 0.456 | -0.004 | 0.987 | 0.160 | 0.500 |
| Basophils(Gpt/L) | 0.073 | 0.768 | 0.159 | 0.504 | 0.210 | 0.375 | 0.412 | 0.071 |
| Alpha-1 globulin (SPEP; %) | -0.028 | 0.91 | 0.259 | 0.270 | 0.019 | 0.937 | 0.381 | 0.097 |
| Alpha-2 globulin (SPEP; %) | 0.174 | 0.476 | 0.287 | 0.221 | 0.073 | 0.759 | 0.314 | 0.178 |
| Albumin (SPEP; %) | -0.056 | 0.82 | -0.323 | 0.164 | -0.213 | 0.367 | -0.307 | 0.188 |
| Beta Globulin (SPEP; %) | -0.066 | 0.788 | 0.191 | 0.420 | 0.255 | 0.279 | 0.041 | 0.865 |
| Gamma Globulin (SPEP;%) | 0.055 | 0.824 | 0.079 | 0.742 | 0.075 | 0.755 | 0.127 | 0.593 |
| C-reactive protein (mg/L) | 0.467 | 0.038 | 0.164 | 0.477 | -0.165 | 0.487 | 0.112 | 0.629 |
| Eosinophils (Differential count; %). | 0.764 | **<0.001** | 0.066 | 0.783 | -0.115 | 0.630 | -0.111 | 0.641 |
| Eosinophils (Gpt/L) | 0.642 | **0.003** | 0.111 | 0.642 | -0.105 | 0.661 | -0.070 | 0.768 |
| Red-cell count | 0.276 | 0.239 | -0.546 | **0.011** | -0.422 | 0.064 | -0.502 | 0.020 |
| Hemoglobin (mmol/L) | 0.209 | 0.377 | -0.172 | 0.455 | -0.357 | 0.122 | -0.435 | 0.049 |
| Hematocrit | 0.203 | 0.392 | -0.187 | 0.417 | -0.367 | 0.111 | -0.455 | 0.038 |
| Immunoglobulin A (g/L) | -0.168 | 0.492 | 0.242 | 0.305 | 0.335 | 0.149 | -0.037 | 0.877 |
| Immunoglobulin E (kU/L) | 0.712 | **<0.001** | -0.008 | 0.971 | -0.012 | 0.962 | -0.087 | 0.706 |
| Immunoglobulin G (g/L) | 0.096 | 0.696 | 0.221 | 0.348 | -0.047 | 0.843 | 0.185 | 0.434 |
| Immunoglobulin M (g/L) | -0.454 | 0.051 | 0.080 | 0.738 | 0.105 | 0.660 | 0.436 | 0.054 |
| White-cell count | -0.524 | 0.018 | 0.136 | 0.557 | 0.014 | 0.954 | 0.197 | 0.392 |
| Lymphocytes (Differential count; %) | 0.095 | 0.7 | 0.087 | 0.715 | 0.070 | 0.770 | -0.032 | 0.893 |
| Lymphocytes (Gpt/L) | -0.27 | 0.264 | 0.238 | 0.313 | 0.160 | 0.499 | 0.144 | 0.546 |
| Mean corpuscular hemoglobin (fmol) | -0.024 | 0.921 | 0.402 | 0.071 | -0.036 | 0.879 | -0.047 | 0.841 |
| Mean corpuscular hemoglobin concentration (mmol/L) | 0.149 | 0.531 | -0.036 | 0.877 | -0.205 | 0.386 | -0.186 | 0.421 |
| Mean corpuscular volume (fL) | -0.1 | 0.676 | 0.492 | 0.023 | 0.044 | 0.855 | 0.039 | 0.865 |
| Monocytes (Differential count; %) | 0.339 | 0.156 | -0.106 | 0.655 | -0.050 | 0.833 | -0.177 | 0.454 |
| Monocytes (Gpt/L) | -0.164 | 0.503 | 0.035 | 0.884 | -0.069 | 0.773 | -0.019 | 0.937 |
| Neutrophils (Gpt/L) | -0.431 | 0.066 | 0.042 | 0.861 | -0.051 | 0.832 | 0.146 | 0.538 |
| Neutrophils (Differential count; %) | -0.252 | 0.297 | -0.079 | 0.742 | -0.040 | 0.866 | 0.067 | 0.780 |
| Procalcitonin (ng/mL) | -0.161 | 0.51 | 0.288 | 0.218 | 0.165 | 0.488 | 0.000 | 1.000 |
| Red Blood Cell Distribution Width (%) | -0.077 | 0.755 | -0.310 | 0.184 | 0.071 | 0.765 | -0.072 | 0.764 |
| Platelet count (Gpt/L) | 0.462 | 0.04 | 0.383 | 0.086 | 0.376 | 0.102 | 0.315 | 0.164 |
| Proteins, total (g/Ll) | -0.129 | 0.6 | 0.335 | 0.149 | 0.129 | 0.587 | 0.308 | 0.186 |

*associations among serologic parameter and GBI/STBI outcome were examined via Pearson product-moment correlations; r= correlation coefficient; significant p values (<0.0125) in bold; IU = International Unit; Gpt/l = 109 cells per liter; SPEP = Serum protein electrophoresis
