# Supplementary material for: Clinical significance and diagnostic usefulness of serologic markers for improvement of outcome of tonsillectomy in adults with chronic tonsillitis
Source: J Negat Results Biomed. 2013 Jul 1;12:11. doi: 10.1186/1477-5751-12-11 (PMC3701599; doi:10.1186/1477-5751-12-11)
Supplement: Additional file 2: Table S2 — Correlation between of preoperative serology (T-1) and functional outcome at T180 in the group of patients with peritonsillar abscess (PTA); r and p-values*. [file 1477-5751-12-11-S2.doc]

**Additional file 2: Table S2. Correlation between of preoperative serology (T-1) and functional outcome at T180 in the group of patients with peritonsillar abscess (PTA); r and p-values***

| Serology at T-1 | GBI  T180  Social | | GBI  T180  Physical | | SBTI  T180  Resources | | SBTI  T180  Benefit | |
| --- | --- | --- | --- | --- | --- | --- | --- | --- |
| Parameter | r | p | r | p | r | p | r | p |
| Antistreptolysin O titer (IU/mL) | 0.423 | 0.103 | 0.059 | 0.827 | -0.143 | 0.626 | -0.079 | 0.772 |
| Basophils (Differential count; %) | -0.243 | 0.382 | -0.051 | 0.856 | -0.275 | 0.363 | 0.076 | 0.788 |
| Basophils(Gpt/L) | -0.228 | 0.413 | 0.180 | 0.521 | -0.391 | 0.187 | -0.085 | 0.763 |
| Alpha-1 globulin (SPEP; %) | 0.320 | 0.244 | -0.371 | 0.174 | 0.137 | 0.655 | -0.495 | 0.060 |
| Alpha-2 globulin (SPEP; %) | 0.116 | 0.682 | -0.464 | 0.081 | -0.100 | 0.746 | -0.348 | 0.204 |
| Albumin (SPEP; %) | -0.328 | 0.233 | 0.204 | 0.467 | 0.235 | 0.440 | 0.249 | 0.370 |
| Beta Globulin (SPEP; %) | 0.248 | 0.372 | -0.195 | 0.486 | -0.030 | 0.924 | -0.366 | 0.180 |
| Gamma Globulin (SPEP;%) | 0.084 | 0.766 | 0.153 | 0.585 | -0.223 | 0.464 | 0.253 | 0.363 |
| C-reactive protein (mg/L) | 0.178 | 0.526 | 0.047 | 0.869 | 0.417 | 0.157 | -0.236 | 0.396 |
| Eosinophils (Differential count; %). | 0.065 | 0.818 | -0.244 | 0.381 | -0.280 | 0.354 | -0.022 | 0.938 |
| Eosinophils (Gpt/lL | -0.083 | 0.768 | -0.145 | 0.605 | -0.542 | 0.056 | -0.229 | 0.413 |
| Red-cell count | -0.409 | 0.130 | 0.168 | 0.550 | 0.292 | 0.332 | 0.507 | 0.054 |
| Hemoglobin (mmol/L) | -0.422 | 0.117 | 0.321 | 0.244 | 0.291 | 0.334 | 0.556 | 0.032 |
| Hematocrit | -0.422 | 0.117 | 0.295 | 0.286 | 0.287 | 0.341 | 0.555 | 0.032 |
| Immunoglobulin A (g/L) | -0.308 | 0.246 | 0.326 | 0.217 | 0.012 | 0.969 | 0.045 | 0.868 |
| Immunoglobulin E (kU/L) | -0.090 | 0.761 | -0.036 | 0.903 | -0.313 | 0.298 | -0.216 | 0.459 |
| Immunoglobulin G (g/L) | 0.052 | 0.847 | 0.310 | 0.242 | -0.188 | 0.520 | 0.283 | 0.288 |
| Immunoglobulin M (g/L) | 0.394 | 0.131 | -0.050 | 0.855 | -0.339 | 0.236 | -0.186 | 0.490 |
| White-cell count | -0.122 | 0.664 | 0.228 | 0.413 | -0.510 | 0.075 | -0.210 | 0.452 |
| Lymphocytes (Differential count; %) | 0.217 | 0.437 | -0.107 | 0.705 | 0.203 | 0.505 | 0.279 | 0.313 |
| Lymphocytes (Gpt/L) | 0.043 | 0.880 | 0.119 | 0.672 | -0.267 | 0.377 | 0.022 | 0.938 |
| Mean corpuscular hemoglobin (fmol) | -0.062 | 0.825 | 0.562 | 0.029 | 0.022 | 0.944 | 0.200 | 0.474 |
| Mean corpuscular hemoglobin concentration (mmol/L) | -0.173 | 0.537 | 0.269 | 0.332 | 0.119 | 0.699 | 0.246 | 0.377 |
| Mean corpuscular volume (fL) | 0.104 | 0.712 | 0.501 | 0.057 | -0.124 | 0.686 | 0.015 | 0.958 |
| Monocytes (Differential count; %) | -0.353 | 0.197 | 0.200 | 0.476 | 0.240 | 0.430 | -0.017 | 0.952 |
| Monocytes (Gpt/L) | -0.324 | 0.239 | 0.290 | 0.295 | -0.102 | 0.740 | -0.160 | 0.569 |
| Neutrophils (Gpt/L) | -0.097 | 0.732 | 0.177 | 0.528 | -0.519 | 0.069 | -0.227 | 0.416 |
| Neutrophils (Differential count; %) | -0.029 | 0.920 | 0.023 | 0.936 | -0.232 | 0.445 | -0.211 | 0.450 |
| Procalcitonin (ng/mL) | 0.222 | 0.427 | 0.048 | 0.865 | 0.550 | 0.052 | -0.013 | 0.963 |
| Red Blood Cell Distribution Width (%) | 0.014 | 0.959 | -0.046 | 0.871 | 0.363 | 0.222 | 0.337 | 0.220 |
| Platelet count (Gpt/L) | 0.914 | -0.030 | 0.010 | 0.972 | -0.676 | **0.011** | -0.165 | 0.557 |
| Proteins, total (g/L) | 0.091 | 0.736 | 0.343 | 0.193 | -0.138 | 0.639 | 0.215 | 0.423 |

*associations among serologic parameter and GBI/STBI outcome were examined via Pearson product-moment correlations; r= correlation coefficient; significant p values (<0.0125) in bold; IU = International Unit; Gpt/L = 109 cells per liter; SPEP = Serum protein electrophoresis
