# Supplementary material for: Clinical significance and diagnostic usefulness of serologic markers for improvement of outcome of tonsillectomy in adults with chronic tonsillitis
Source: J Negat Results Biomed. 2013 Jul 1;12:11. doi: 10.1186/1477-5751-12-11 (PMC3701599; doi:10.1186/1477-5751-12-11)
Supplement: Additional file 3: Table S3 — Changes of serology within each group of patient between T-1 and T5. [file 1477-5751-12-11-S3.doc]

**Additional file: Table S3**

| **Table S3. Changes of serology within each group of patient between T-1 and T5** | | | | | |
| --- | --- | --- | --- | --- | --- |
| Parameter | Chronic tonsillitis | | Peritonsillar abscess | | Significant change of parameter |
|  | Increase or decrease | p* | Increase or decrease | p* | Same or opposite direction** |
| Antistreptolysin O titer (IU/mL) | Decrease | 0.084 | Increase | 0.105 |  |
| Basophils (Differential count; %) | Decrease | 0.280 | Increase | **<0.0001** | opposite |
| Basophils(Gpt/L) | Decrease | 0.234 | Increase | 0.337 |  |
| Alpha-1 globulin (SPEP; %) | Increase | **<0.0001** | Decrease | 0.060 | opposite |
| Alpha-2 globulin (SPEP; %) | Increase | **<0.0001** | Decrease | 0.975 | opposite |
| Albumin (SPEP; %) | Decrease | **<0.0001** | Increase | 0.589 | opposite |
| Beta Globulin (SPEP; %) | Increase | **0.010** | Decrease | **0.007** | opposite |
| Gamma Globulin (SPEP;%) | Decrease | **<0.0001** | Increase | 0.056 | opposite |
| C-reactive protein (mg/L) | Increase | **0.001** | Decrease | **<0.0001** | opposite |
| Eosinophils (Differential count; %). | Increase | 0.229 | Increase | **<0.0001** | same |
| Eosinophils (Gpt/L) | Increase | 0.054 | Increase | **<0.0001** | same |
| Red-cell count | Decrease | **<0.0001** | Decrease | **0.038** | same |
| Hemoglobin (mmol/L) | Decrease | **<0.0001** | Decrease | **0.016** | same |
| Hematocrit | Decrease | **<0.0001** | Decrease | **0.022** | same |
| Immunoglobulin A (g/L) | Decrease | **0.043** | Increase | 0.145 | opposite |
| Immunoglobulin E (kU/L) | Increase | 0.063 | Decrease | 0.143 |  |
| Immunoglobulin G (g/L) | Decrease | **<0.0001** | Increase | 0.565 | opposite |
| Immunoglobulin M (g/L) | Decrease | **0.008** | Increase | 0.502 | opposite |
| White-cell count | Increase | 0.773 | Decrease | **<0.0001** | opposite |
| Lymphocytes (Differential count; %) | Decrease | **0.003** | Increase | **<0.0001** | opposite |
| Lymphocytes (Gpt/L) | Decrease | **0.005** | Increase | **0.031** | opposite |
| Mean corpuscular hemoglobin (fmol) | Decrease | **0.033** | Decrease | **0.032** | same |
| Mean corpuscular hemoglobin concentration (mmol/L) | Decrease | 0.524 | Decrease | **0.389** | same |
| Mean corpuscular volume (fL) | Decrease | 0.182 | Decrease | 0.130 |  |
| Monocytes (Differential count; %) | Increase | 0.293 | Decrease | 0.200 |  |
| Monocytes (Gpt/L) | Increase | 0.413 | Decrease | **<0.0001** | opposite |
| Neutrophils (Gpt/L) | Increase | 0.336 | Decrease | **<0.0001** | opposite |
| Neutrophils (Differential count; %) | Increase | **0.015** | Decrease | **<0.0001** | opposite |
| Procalcitonin (ng/mL) | Decrease | **0.003** | Decrease | **0.001** | same |
| Red Blood Cell Distribution Width (%) | Decrease | **0.005** | Decrease | **<0.0001** | same |
| Platelet count (Gpt/L) | Decrease | 0.367 | Increase | **<0.0001** | opposite |
| Proteins, total (g/L) | Decrease | **<0.0001** | Decrease | **0.025** | same |

*p = p value due to Wilcoxon test; significant p values (<0.05) in bold; IU = International Unit; Gpt/L = 10^9^ cells per liter; SPEP = Serum protein electrophoresis; **in green = change in opposite direction, red = in same direction, dark color = significant change in both groups; light color = significant change in one group.
